# Biophysical and Biological Mechanisms of Tumor Treating Fields in Glioblastoma

**DOI:** 10.26502/jcsct.5079249

**Published:** 2024-08-19

**Authors:** Jeremy Pan, Tony Eskandar, Zubair Ahmed, Devendra K. Agrawal

**Affiliations:** 1 Department of Translational Research, College of Osteopathic Medicine of the Pacific, Western University of Health Sciences, Pomona CA 91766, USA

**Keywords:** Angiogenesis, Brain tumor, Glioblastoma, Glioma, MacDonald criteria, Mitotic spindle development, Optune, Radiotherapy, Response assessment in neuro-oncology criteria (RANO), Stupp protocol, Tumor response criteria, Tumor treated fields

## Abstract

Glioblastoma (GBM) is one of the most aggressive forms of brain cancer that presents with a median survival rate of 14–30 months and along with a discouraging five-year survival rate of 4–5%. Standard treatment of newly diagnosed GBM, also known as the Stupp protocol, includes a maximally safe surgical resection followed by radiation and chemotherapy. Despite these treatment regimens, recurrence is almost inevitable, emphasizing the need for new therapies to combat the aggressive nature of GBMs. Tumor Treating Fields (TTFs) are a relatively new application to the treatment of GBMs, and results have been promising with both progression-free survival and overall survival when TTFs have been used in combination with temozolomide. This article critically reviews the biophysical and biological mechanisms of TTFs, their clinical efficacy, and discusses the results in clinical trials, including EF-11 and EF-14. Both trials have demonstrated that TTFs can enhance progression free survival and overall survival without compromising quality of life or causing severe adverse effects. Despite the high cost associated with TTFs and the need for further analysis to determine the most effective ways to integrate TTFs into GBM treatments, TTFs represent a significant advancement in GBM therapy and offer hope for improved patient prognosis.

## 1. Introduction

Globally, Glioblastoma (GBM), a subset of glioma, presents itself as a rapidly growing and aggressive brain tumor, leading to an extremely poor prognosis with a median survival currently ranging from 14 to 30 months depending on the molecular subtypes the tumor possesses [1–4]. Common symptoms in patients diagnosed with GBM include increased intracranial pressure, headaches, neurological deficits, and epilepsy [4]. Currently, there are approximately 3 to 5 cases per 100,000 persons diagnosed each year worldwide [5, 6] with a 5-year survival rate at a dismal 4–5% [7–9]. Gliomas are graded on a universal scale from I to IV. Grades I and II are considered low-grade, consisting of slow-growing benign tumors found mainly in the pediatric population. Grade III is considered high-grade, consisting of astrocytoma and oligodendroglioma. Grade IV gliomas are known as glioblastoma (GBM) and these are the fastest-growing tumors with vascular proliferation and necrosis. With such a low prognosis, clinicians and scientists all over the world still strive for a treatment method that is effective and can preserve a decent quality of life. Tumor Treating Fields (TTFs) are a relatively new treatment modality and usage has remained infrequent, with only 3–12% of newly diagnosed GBM (ndGBM) patients and 0–16% recurrent GBM (rGBM) patients utilizing TTFs [10, 11]. Although many physicians have been doubtful about the clinical value and research studies of TTFs, recent research findings have shown that TTFs exhibit a variety of biophysical and biological effects such as antimitotic effects, cell migration, increased blood brain barrier penetration, and many more that could make it a useful therapy for many cancers [12]. In the phase 3 EF-11 trial of rGBM, TTF monotherapy vs physicians’ choice chemotherapy showed comparable survival benefits, 6.6 vs 6.0 months, respectively [13, 14]. Furthermore, in the phase 3 EF-14 clinical trial for GBMs, it was discovered that the median PFS in ndGBM patients undergoing TTF treatment combined with temozolomide (TMZ) was longer than those treated with TMZ alone, 7.1 months and 4.0 months, respectively [14–16]. Overall survival was improved with TTF + TMZ as well at 19.6 months versus TMZ only at 16.0 months [9, 13, 14, 15, 17, 18]. It was also found that the two-year survival rate in ndGBM patients treated with TTF + TMZ was 14% higher than those treated with TMZ alone [15]. Therefore, incorporating TTFs in conjunction with the standard treatment of GBM does show some promise as a strong consideration in GBM cases. This review article analyzed the current statistics, definitions, and treatments of GBM. It also seeks to evaluate the mechanism of action and efficacy of Tumor Treating Fields therapy for GBM, including associated outcomes and complications.

## 2. Diagnosis and treatment of glioblastoma – an overview

When a patient is radiologically diagnosed with GBM, the current standard of care treatment plan, also known as the Stupp protocol, includes a maximally safe surgical resection with post-operative concurrent chemoradiation and chemotherapy [13, 19]. The extent of resection of the patient’s tumor is calculated using MRI with contrast-enhanced T1 imaging [20]. By looking at the transverse slice and calculating tumor volume, the physician can determine the extent of resection using the following equation: (preoperative tumor volume – postoperative tumor volume)/preoperative tumor volume. A percentage of tumor removed that is greater than 95% is defined as a gross-total resection (GTR) [20], and a percentage lower than 95% is defined as a subtotal resection (STR) [21]. Following surgical resection, radiotherapy and temozolomide chemotherapy is administered, where 60 Gray (Gy) of focal radiotherapy is given in 2 Gy fractions over a period of 6 weeks with a 28-day, daily dosing regimen of Temozolomide (TMZ) [14, 22].

Recurrence of tumor after surgery, radiotherapy, and chemotherapy is very common and considered inevitable [23]. The median time to tumor recurrence is found to be 32–36 weeks, and approximately 90% of GBM patients will experience recurrence within 2 years of initial GBM diagnosis [24]. Many common symptoms of tumor recurrence include increased onset of nausea, weakness, seizures, and headaches of varying severity as well as neurocognitive symptoms including aphasia, vision loss, and gait instability [13]. For both recurrent or progressive GBMs, there is no standard of care treatment plan established due to a lack of appropriate research and molecular variability with each tumor [19, 25, 26]. Only a minority of patients qualify for re-resection or reirradiation, and others can achieve progression-free survival (PFS) for 6 months with TMZ dosing regimens, reirradiation, bevacizumab, and palliative care [13, 19, 27]. Even with these options, long-term survival rates of GBM patients have remained low, so TTFs have emerged as a new methodology in the potential reversal of this trend.

## 3. Criteria for tumor response

Tumor response criteria have also been developed to determine the efficacy of a treatment in stabilizing or reducing bidimensional tumor measurements while on treatment [28]. Many scientists and clinicians utilize the Macdonald criteria, which has since been superseded by the Response Assessment in Neuro-Oncology (RANO) criteria (Table 1), both of which place tumor response into four categories: complete response (CR), partial response (PR), stable disease (SD), and progressive disease (PD) (Table 2) [28, 29]. Progressive disease is defined as a greater than 25% volume increase of the primary enhancing lesions while the patient is taking stable or increasing doses of corticosteroids, growth of new lesions, and any significant increase in non-enhancing lesions. Clinical neurological decline is also associated with progressive disease.

## 4. Overview of tumor treating field

TTFs are delivered using a system called Optune (Novocure), which includes four transducer arrays, an electric field generator, and a power source. Since GBM tumors are in the brain, patients using Optune will shave their head to allow for transducers to be attached at the scalp in pairs. This allows optimal contact to the scalp and positioning relative to the tumor size and location. Each transducer has 9 ceramic discs that carry the electric fields produced by the generator via a hydrogel coating across the skin to the tumor. One of the biggest considerations of Optune TTF therapy is the cost. The monthly cost comes out to approximately $21,000, which covers equipment, staffing, and patient and physician support.

## 5. Mechanism of action of tumor treated fields

**Figure 1:**
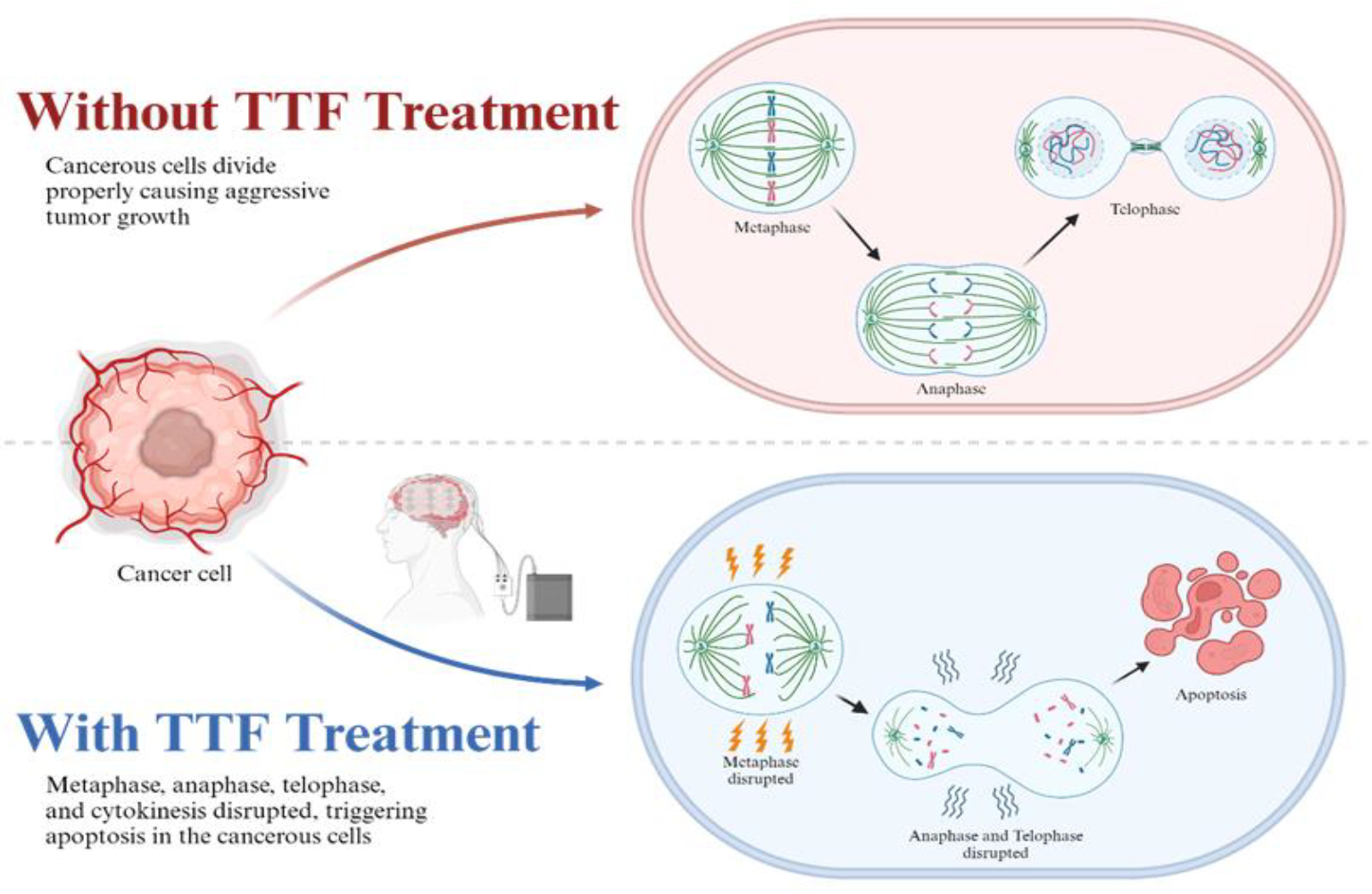
Effects of tumor treated field (TTF) on cell mitosis. Electrical fields disrupt the alignment and mobility of spindle formation, causing improper development downstream leading to cell apoptosis. This figure is created with BioRender.

Tumor Treating Fields are selectively anti-mitotic, and they aim to disrupt cell division of cancerous cells and prevent proper mitotic spindle development through the administration of oscillating electric fields between 100 and 300 kHz with intensities of 1 to 3 V/cm [31, 32, 33]. These electric fields are delivered using a TTF device consisting of four transducer arrays supplied with nine electrodes each that are applied to a patient’s scalp [14]. TTFs target the dipole moments of tubulin subunits. The electric fields generated by the TTF device align microtubules along the alternating electric fields, ultimately disrupting them and interfering with the cell division process [14, 23, 34]. The effects of TTFs vary depending on the stage of cell division. For example, TTFs disrupt the mobility and formation of spindles during metaphase. In anaphase, telophase, and cytokinesis, they cause misalignment and polarity failure of spindles and the contractile ring, leading to a disruption in the cytoplasmic separation and inducing apoptosis of cancerous cells (Figure 1) [13, 35].

Research has shown that 50% of TTF treated cells had disrupted mitosis as opposed to 5% in non-TTF treated cells [14, 34]. Through this mechanism, cellular fragmentation and apoptosis in cancer cells is greatly induced and the permeability of the blood-brain barrier was increased. Tight junction proteins Claudin-5 and ZO-1, in the plasma membrane were disrupted due to the electric fields, causing the integrity of the blood brain barrier to be reduced by 65% temporarily and allowed for large molecules up to 4 kDa to pass through [13, 36]. This temporary disruption helps increase the efficacy of the standard treatment [32]. Furthermore, in 2017, Chang et al. found that TTF treatments worked synergistically with anticancer compound Withaferin A to inhibit the growth of glioblastoma cells [23, 37]. This synergistic effect is due to TTF’s ability to increase the permeability of tumor cell membranes, allowing anticancer compounds like bevacizumab to penetrate more effectively [23, 37]. TTFs have also been shown to promote an adhesive cell phenotype and suppress angiogenesis, thereby inhibiting the spread of cancer cells and reducing the likelihood of metastasis [33]. One significant factor in cancer metastasis is the loss of epithelial differentiation, which occurs due to the decreased E-cadherin-mediated cell junctions and the upregulation of mesenchymal markers such as vimentin [33]. This process, also known as the epithelial-mesenchymal transition (EMT), is unintentionally activated in cancer cells. EMT gives cancer cells the ability to metastasize away from the primary site, evade apoptosis, and contribute to immunosuppression [38–40]. TTFs have been found to reverse this process, increasing the expression of E-cadherin and downregulating mesenchymal markers such as vimentin [33, 38]. Lastly, angiogenesis is the formation of new blood vessels, which is crucial to cancer progression, migration, and invasion. The formation of blood vessels in angiogenesis is mediated by the overexpression of HIF1α, a transcription factor that induces the formation, survival, angiogenesis, and migration of tumors. Vascular endothelial growth factor (VEGF) is the main downstream target of HIF1α for blood vessel formation, and TTFs have been found to decrease the expression of both HIF1α and VEGF in GBM tumors [33, 41]. Therefore, TTFs present a promising approach to limiting tumor growth and spread through multiple mechanism of actions.

## 6. Tumor treated fields: selection criteria and findings of clinical trials

Initially, a small pilot study was conducted to understand the feasibility of TTFs in treating solid tumors. Selection criteria included one or more measurable lesions, a tumor that was accessible by TTFs, and no concomitant antitumor therapy. The trial results were promising; researchers found that TTF treatment at low intensity for 13–46 days was very well tolerated with only a mild, grade 1 skin irritation reported at the electrode placement sites, and out of six subjects, 1 patient had partial response and three had tumor growth arrest during treatment [17, 42]. Efficacy of TTFs was later evaluated in 10 rGBM patients who received TTFs for 280 weeks with no treatment-related adverse events or significant changes in serum chemistry and blood count [17, 43]. Like the pilot study, the rGBM patients reported mild to moderate dermatitis in the area where TTF electrodes were placed, which were treated with topical steroids and replacement of the electrodes [17]. With TTF treatments, it was found that the median time to disease progression extended to 26.1 weeks, and the median overall survival reached 62.2 weeks, both of which are double the medians reported for historical control patients [17, 43]. These results are extremely promising, showing that even with more than 70 months of recurring treatment, TTFs do not cause significant hematological or gastrointestinal toxicities [43]. Two more international, phase III randomized clinical trials were created to further determine the effectiveness of TTFs in GBMs. In the first study, named EF-11, participants were eligible if they had previously been treated with radiotherapy and TMZ and other prior lines of chemotherapy and experienced recurrence or progression during treatment. 237 patients were randomized in a one-to-one ratio where 120 patients received TTF monotherapy and 117 patients were on an active control arm: an oncologist-determined treatment regimen best fit for the patient [17, 18]. It was found that median overall survival (OS) in both groups were relatively similar, with TTF patients at 6.6 months and active control at 6.0 months OS [18]. Although the EF-11 clinical trial did not find a significant superiority of TTF treatment over common chemotherapy regimens, this study further supported the safety and feasibility of TTFs on an international scale. The similarity in OS timelines can also be attributed to the selection of patients, 40% of whom were on their third recurrence and suffered from advanced disease [18]. However, TTF monotherapy did show a higher objective response as compared to the control arm, 14% versus 9.6%, respectively [18, 44, 45]. In the second study, named EF-14, the effectiveness of TTFs in conjunction with standard of care treatments in ndGBM patients was evaluated. Compared to EF-11, there was a statistically significant increase in PFS and OS in patients utilizing a combination of TTFs and TMZ. A total of 695 total patients participated in the clinical trial in a 2:1, TTF plus TMZ regimen versus TMZ control group ratio. In the first data analysis of 315 randomized patients, 210 patients were assigned to the Optune (TTF) plus TMZ group, and 105 patients were assigned to the TMZ monotherapy control group. It was discovered that the OS of the TTF plus TMZ group was significantly higher than the control arm, 19.6 months versus 16.6 months, respectively [17, 18]. As seen with EF-11, there were no severe adverse events attributed to TTF treatment. Only 2% of patients experienced grade 3 skin irritations. After an analysis of all 695 patients enrolled on EF-14, the data presented statistically significant increases PFS and OS for TTF plus TMZ treatment compared to control. PFS was prolonged by 2.7 months and OS was prolonged by 4.8 months [17]. Both EF-11 and EF-14 show that TTFs are not only safe for both ndGBM and rGBM patients, but also have the potential to improve PFS and OS durations when combine with other chemotherapies.

### Conclusion

Glioblastoma (GBM) remains as one of the most aggressive and lethal cancers in the human body with very limited treatment options and grim prognosis. Recurrence of tumor is almost inevitable despite the recent advancements of surgical resections, radiotherapy, and chemotherapy regimens, emphasizing the need for innovative therapeutic strategies such as Tumor Treating Fields (TTFs). Clinical trials, such as EF-11 and EF-14, aiming to discover the benefits and drawbacks of TTFs. The combination treatments with TTFs and TMZ in both ndGBM and rGBM have demonstrated a strong potential to extend the PFS and OS in both patient populations without compromising quality of life or causing severe adverse events, validating the safety and feasibility of TTFs in GBM treatment regimens. This is due to the biophysical mechanisms of TTFs such as their anti-mitotic effects, ability to increase BBB permeability, and suppression of angiogenesis, all of which contribute to its therapeutic efficacy against GBM. However, integrating TTFs into standard of care protocols has current limitations as well – the high costs associated with adhering to the proper TTF treatment regimen is a significant barrier. Additionally, it is important that more clinical trials and studies are conducted to identify the optimal clinical scenarios for TTF use, such as targeting specific biomarkers and determining synergistic potential with emerging therapies aside from the standard of care protocol. TTFs are a strong step forward in the treatment advancement against GBM, and the continued study of TTFs and their mechanisms of actions against GBM is promising in improving the prognosis, quality of life, and survival of all GBM patients.

## Supplementary Material

Supply

## Figures and Tables

**Table 1:** Comparison between MacDonald and RANO response Criteria. Table is modified from Chukwueke and Wen [30].

Criteria	Mac Donald	RANO
**Measurement**	2D Contrast Enhancement	2D Contrast Enhancement+T2/FLAIR
**Progression**	≥25% increase in the product of perpendicular diameter	≥25% increase in the product of perpendicular diameter
**Response**	≥50% decrease in the product of perpendicular diameter	≥50% decrease in the product of perpendicular diameter
**Durability of Response**	Yes (at least 4 week)	Yes (at least 4 week)
**No. of Targets Lesions**	N/A	Up to 5
**T2/FLAIR Evaluation**	Not Evaluated	Evaluated
**Corticosteroid Evaluation**	Yes	Yes
**Clinical Status Evaluation**	Yes	Yes
**Pseudo-progression Evaluation**	No	Yes

**Table 2:** Response Assessment Criteria with MRI and Clinical Status. Table modified from Chukwueke and Wen [30].

Criteria	Complete Response (CR)	Partial Response (PR)	Stable Disease (SD)	Progressive Disease (PD)
**T1 Gadolinium Enhancing Disease**	None	50% Decrease Relative to Baseline	< 50% Decrease to < 25% Increase Relative to Baseline	25% Increase Relative to Baseline
**T2/FLAIR**	Stable or Decreasing	Stable or Decreasing	Stable or Decreasing	Increasing
**New Lesions**	None	None	None	Present
**Corticosteroid Use**	None	Stable or Decreasing	Stable or Decreasing	N/A
**Clinical Status**	Stable or Improving	Stable or Improving	Stable or Improving	Worsening
**Requirements for Response**	All of the Above	All of the Above	All of the Above	Any of the Above
