# Supplementary material for: Biophysical and Biological Mechanisms of Tumor Treating Fields in Glioblastoma
Source: J Cancer Sci Clin Ther. Author manuscript; Available in PMC 2024 Oct 3. (PMC11448370; doi:10.26502/jcsct.5079249)
Supplement: Supply [file NIHMS2019884-supplement-Supply.pdf]

Supporting information

S1 Table: Summary of the IC_{50} values for ETC-206 determined for the 38 primary SelectScreen® kinase hits.

**Kinase Assay**	**IC_{50} (nM)**	**Fold difference (vs. MNK1/2)**
BRAF	>50,000	ND
BRAF V599E (activity)	4,630	54
BRAF V599E (binding)	4,880	57
CDC7/DBF4	5,190	61
CDK11 (Inactive)	2,590	30
CDK8/cyclin C	7,720	90
CSF1R (FMS)	6,300	74
FLT3	1,210	14
FLT3 D835Y	3,760	44
FLT3 ITD	8,580	100
GSG2 (Haspin)	3,180	37
LRRK2 FL	12,400	145
LRRK2 G2019S	13,000	123
LRRK2 G2019S FL	10,500	152
LRRK2 I2020T	8,980	105
LRRK2 R1441C	23,100	270
MAP2K1 (MEK1) S218D S222D	4,950	58
MAP2K2 (MEK2)	4,170	49
MAP4K2 (GCK)	8,020	94
MKNK1 (MNK1)	64	1
MKNK2 (MNK2)	86	1
NEK4	27,700	323
NLK	1,660	19
NTRK1 (TRKA)	2,550	30
NTRK2 (TRKB)	2,370	28
NTRK3 (TRKC)	1,370	16
PDGFRA V561D	4,340	51
PI4KB (PI4K beta)	997	12
PIK3C2B (PI3K-C2 beta)	49,700	580
PIK3CA/PIK3R1 (p110 alpha/p85 alpha)	9,500	111
PIK3CD/PIK3R1 (p110 delta/p85 alpha)	>50,000	ND
PTK6 (Brk)	6,710	78
RIPK2	610	7
RIPK3	11,000	128
STK16 (PKL12)	6,020	70
STK17A (DRAK1)	3,830	45
STK17B (DRAK2)	3,940	46
TXK	4,240	49

S2 Table: Summary of the IC_{50} values for ETC-206 determined using the CellTiter-Glo® Luminescent Cell Viability Assay 72 h post-treatment for a panel of 71 cell lines, comprising liquid tumor cell lines, lymphoma, myeloma, and non-cancerous human cell lines derived from PBMCs.

**Cell Line**	**Cell type**	**IC_{50} (μM)**
I9.2	Acute T-cell leukemia	6.38
J45.01	Acute T-cell leukemia	11.74
P116	Acute T-cell leukemia	13.22
J.RT3-T3.5	Acute T-cell leukemia	14.84
Jurkat	Acute T-cell leukemia	17.98
J.gamma.1	Acute T-cell leukemia	23.65
D1.1	Acute T-cell leukemia	>50.00
8E5	ALL	15.32
RS4;11	ALL	31.31
KE-37	ALL	32.22
Molt-4	ALL	39.12
MOLT-3	ALL	26.64
CEM-CM3	ALL (juvenile)	14.90
CEM/C2	ALL (juvenile)	24.04
CCRF-HSB-2	ALL (juvenile)	48.67
SUP-B15	ALL Ph^+ (juvenile)	38.90
Kasumi-1	AML	18.90
Loucy	AML	33.95
AML-193	AML	>50.00
MV-4-11	AML (biphenotypic B-myelomonocytic)	15.02
EOL-1	AML (eosinophilic)	21.43
TF-1	AML (erythroid)	48.74
THP-1	AML (monocytic)	48.51
BDCM	AML (monocytic, M5a)	32.21
CESS	AML (myelomonocytic)	3.15
HL60	AML (promyelocytic)	48.50
Clone 15 HL-60	AML (promyelocytic)	49.71
BC-2	B-cell lymphoma	35.31
BC-1	B-cell lymphoma	23.67
MC116	B-cell lymphoma (undifferentiated)	>50.00
RPMI 7666	B-lymphoblast (non-cancer)	29.44
GK-5	B-lymphoblast (non-cancer)	3.36
AHH-1	B-lymphoblast (non-cancer)	26.85
NAMALWA	Bukitt's lymphoma	1.60
ST486	Bukitt's lymphoma	16.03
EB1	Bukitt's lymphoma	16.22

S1 Figure: Western blots for p-eIF4E inhibition in different tissues in naïve ICR and tumor-bearing SCID mice after single-dose treatment.

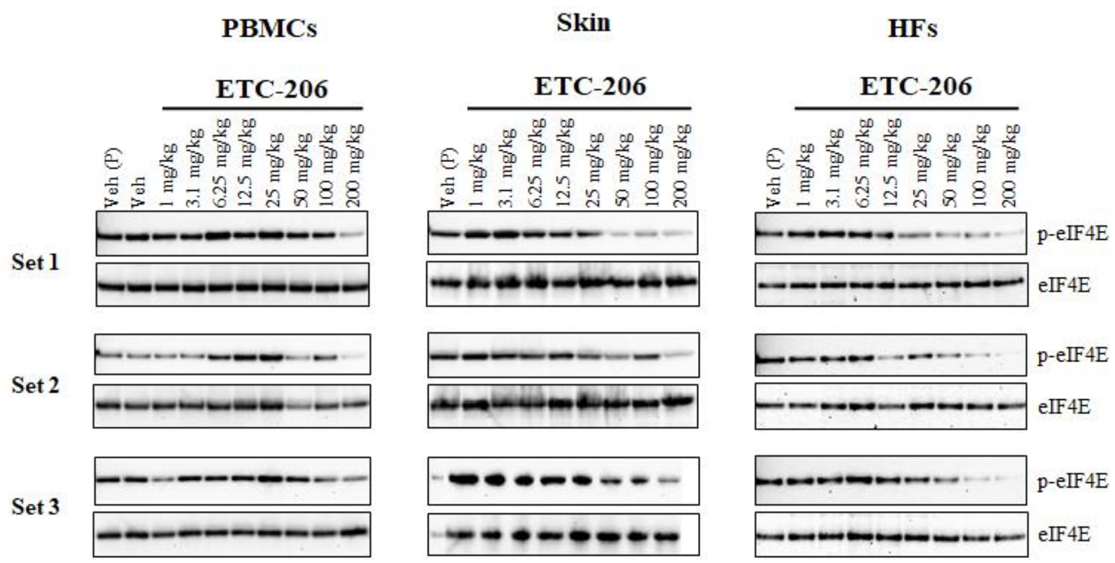

(A) Determination of relative p-eIF4E/eIF4E levels in surrogate tissues harvested from female ICR mice treated for 2 h with ETC-206 as indicated. Tissue samples from surrogate tissues were lyzed, separated on Bis-Tris protein gels, and analyzed via Western blot analysis with antibodies as indicated; protein loading was 10 µg of total protein per lane for PBMCs, 8 µg of total protein per lane for skin, and 4 µg of total protein per lane for HFs. Veh, vehicle-treated animal, Veh (P), pooled samples from 3 vehicle-treated animals. The corresponding densitometry analysis is shown in Figure 3B. "Set 1", "Set 2", etc. represent identically treated sample sets from replicate animals.

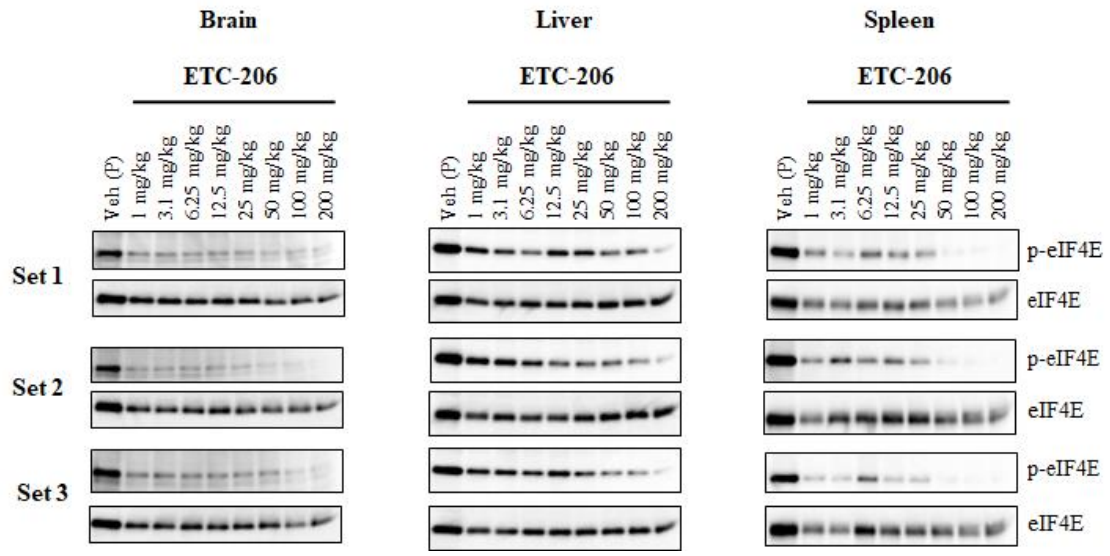

(B) Brain, liver, and spleen samples (5 µg of total protein was loaded per lane for brain, liver, and spleen) were harvested from the same animals as shown in (A) and analyzed by Western blot as described above.

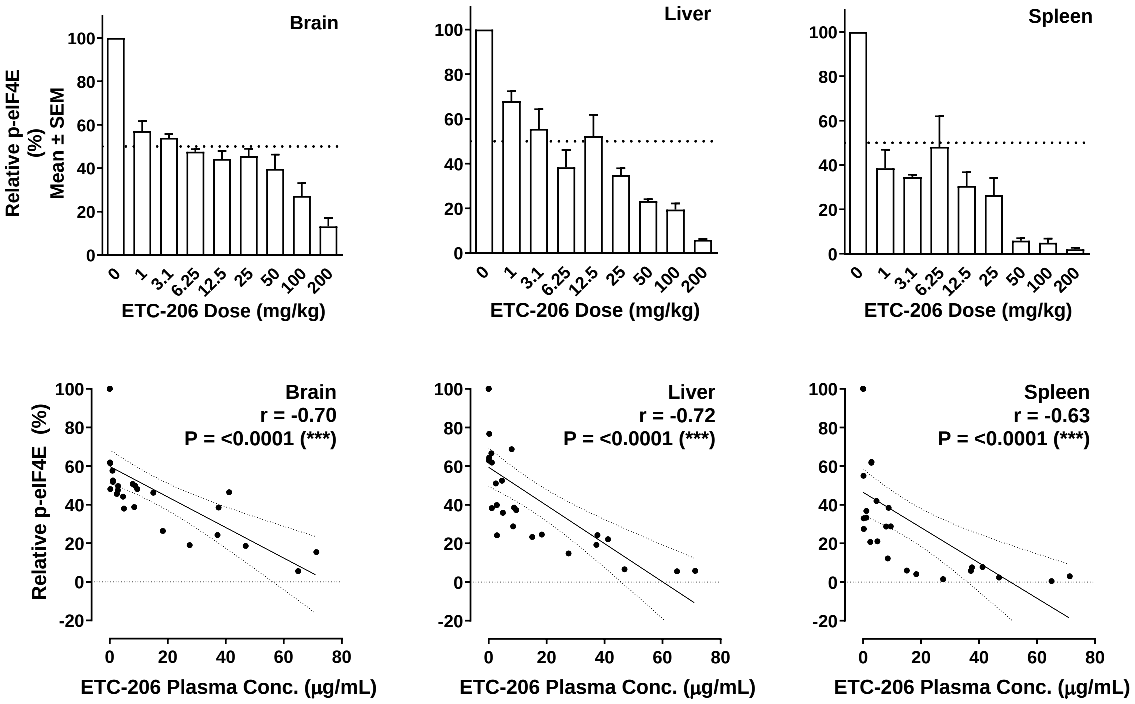

(C) Upper panels: relative p-eIF4E levels were determined from the Western blots shown in (B) and analyzed by densitometry. Lower panels: correlation between ETC-206 plasma concentrations and relative p-eIF4E levels (solid lines: linear correlation, dotted lines: 95% confidence intervals; Pearson's r and corresponding P values are inserted).

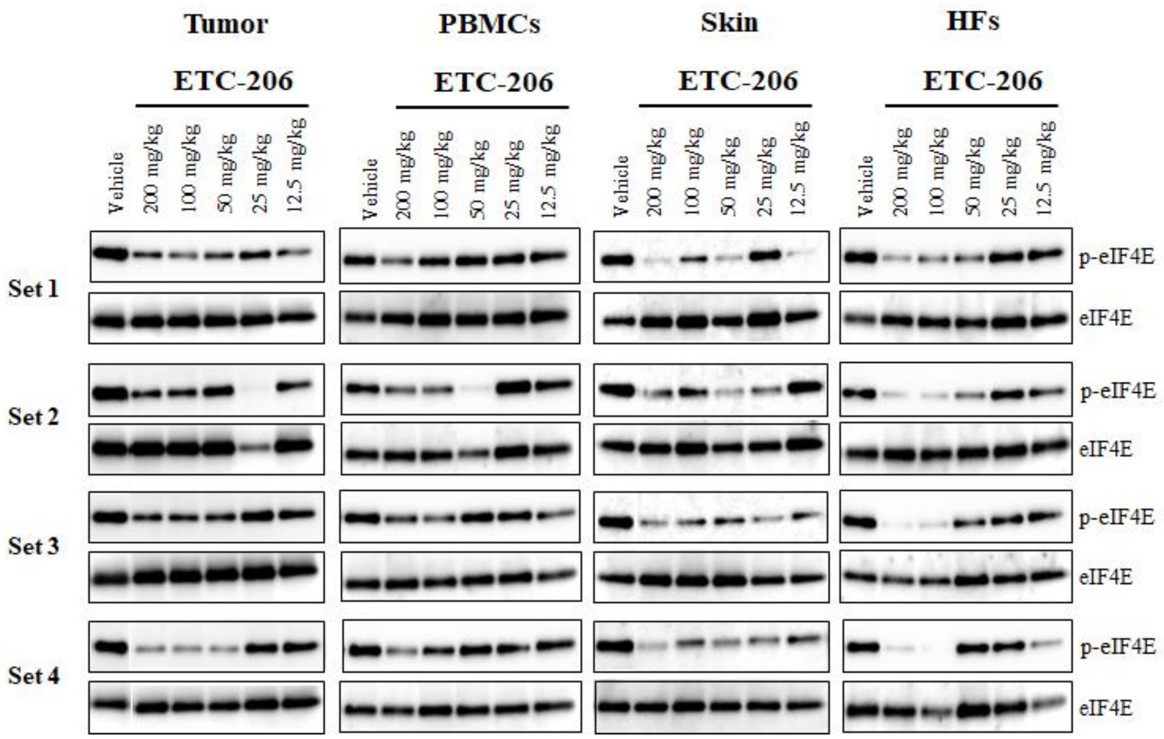

(D) Determination of relative p-eIF4E/eIF4E levels in tumor and surrogate tissues harvested from tumor-bearing SCID mice treated with ETC-206 as indicated and analyzed by Western blot as described above (5 µg of total protein per lane for tumor tissue, 10 µg of total protein per lane for PBMCs, 5 µg of total protein per lane for skin, and 3.5 µg total of protein per lane for HFs). The corresponding densitometry analysis is shown in Figure 3C.

S2 Figure: Western blots showing effects of a single-dose of ETC-206 on relative p-eIF4E levels in PBMCs, hair follicles, and skin of human volunteers.

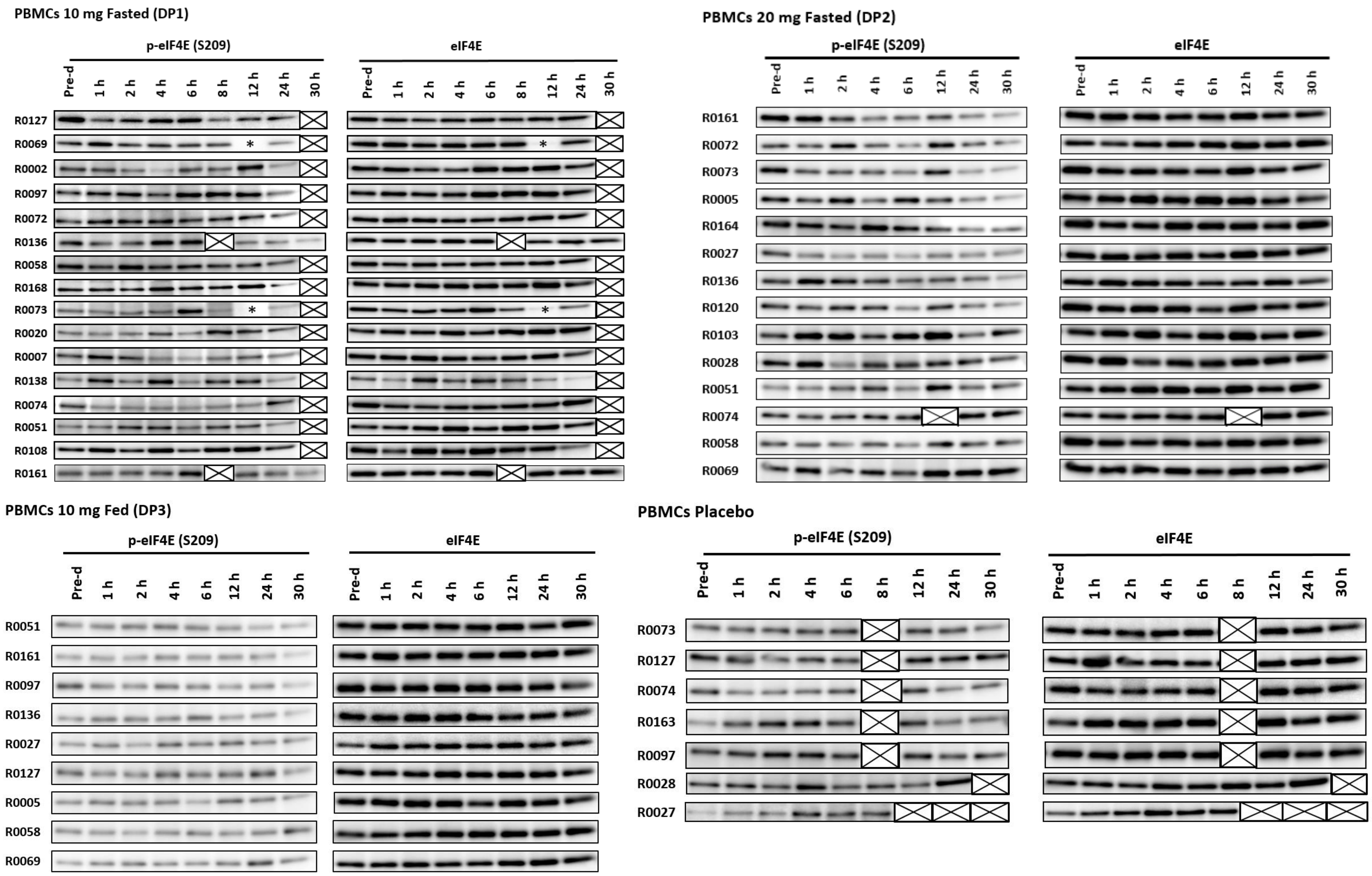

(A) Western blots from PBMCs for the data set shown in Figure 4 are shown. Refer to respective figure legend for study/experimental details. Asterisks (*) indicate samples that were partially lost due to cracked vials and values that therefore have not been included, crossed boxes (X) indicate samples that were not collected due to clinical protocol specifications, or outliers.

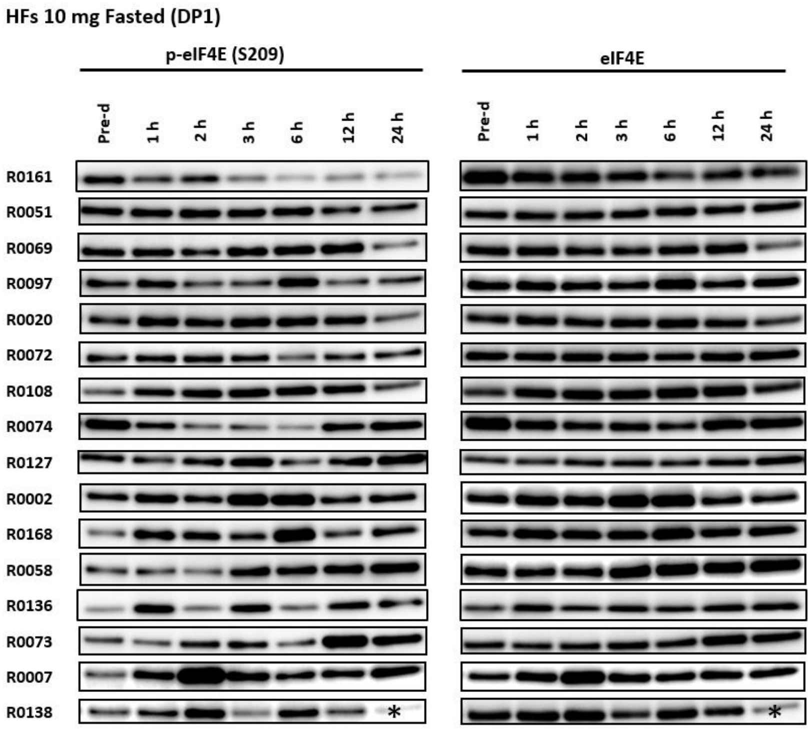

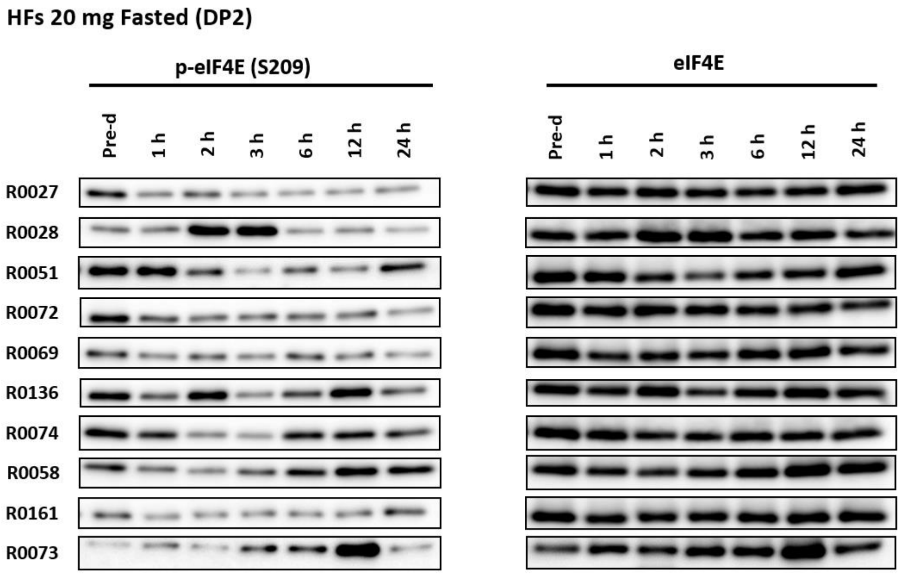

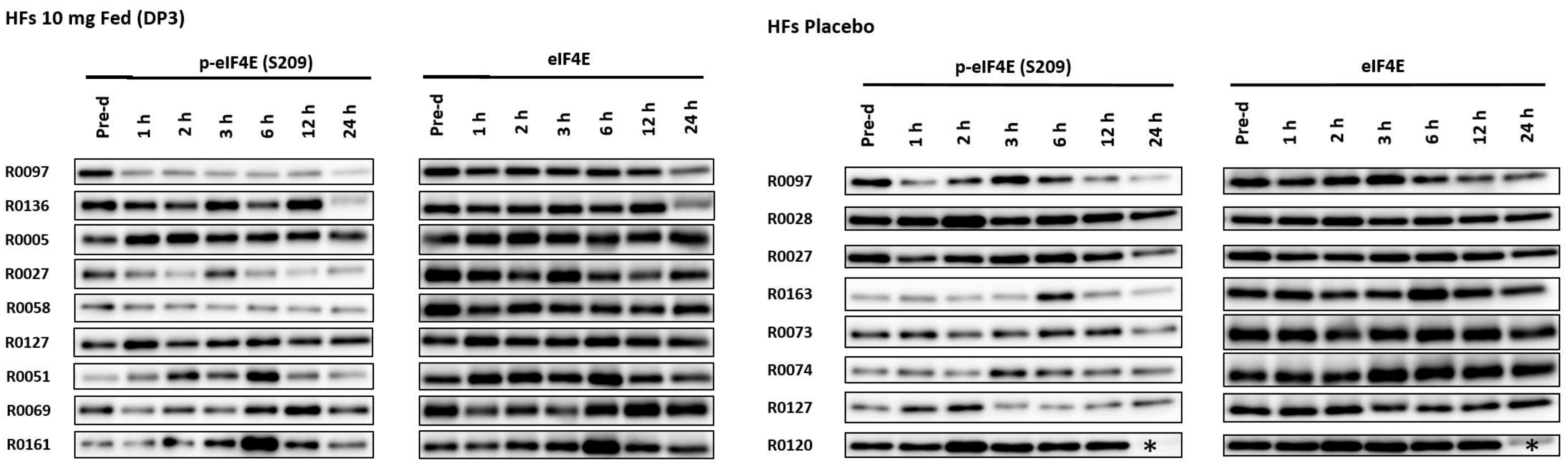

(B) Western blots from HFs for the data set shown in Figure 5 are shown. Refer to respective figure legend for study/experimental details. Asterisks (*) indicate samples that were partially lost due to cracked vials and values that therefore have not been included.

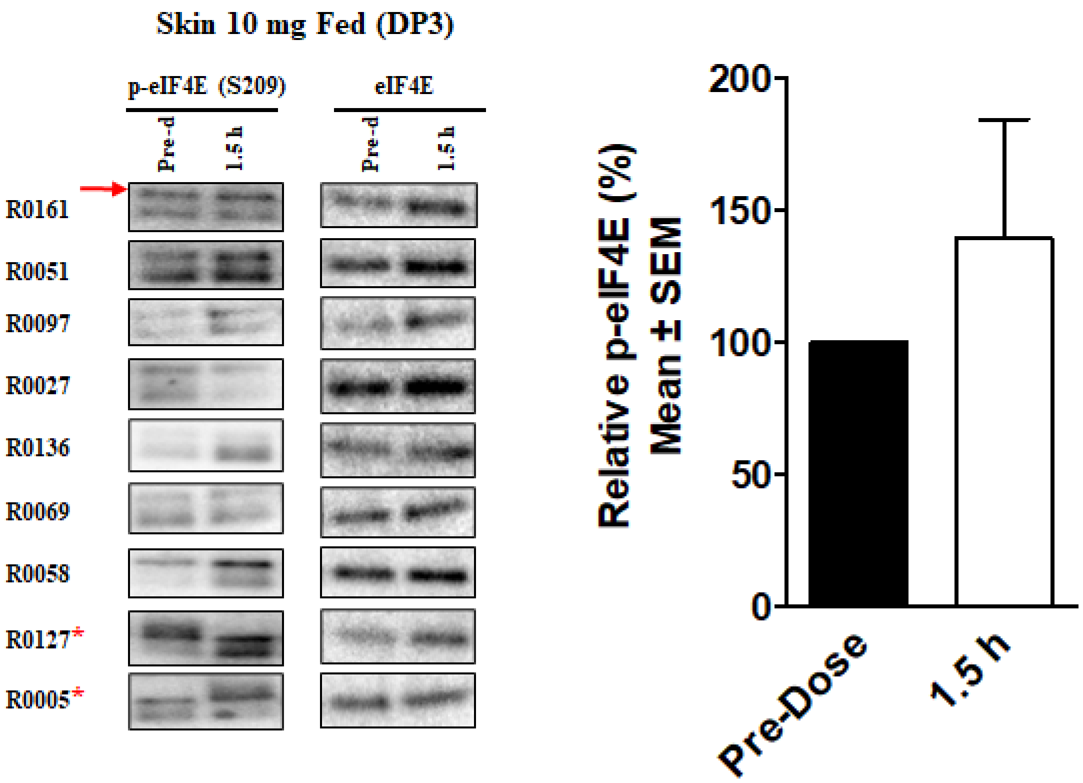

(C) Effect of a single dose of 10 mg ETC-206 on the relative p-eIF4E levels in skin samples from human HVs. Subjects received a single dose of 10 mg in the fed state after a high-fat meal. Skin was collected by punch-biopsy from the back from each subject at pre-dose and 1.5 h post-dose (n=9). Western blot results are shown on the left, * indicates samples excluded from densitometry analysis due to incorrect MW; in human skin, a double band is detected of which only the upper band corresponds to the correct molecular weight (red arrow). Mean values of relative p-eIF4E levels at pre- and post-dose are shown on the right (n=7).

Supplementary Methods

Mouse blood plasma preparation

Whole blood from mice was obtained by cardiac puncture or retro-orbital bleeding according to local animal welfare regulations, using K_2EDTA as an anticoagulant. For non-terminal bleedings, ~0.2 mL blood was collected in 300 µL Microvette® tubes (Cat. #CB300 K2E; Sarstedt, Germany). Blood was centrifuged at 2,000 rpm for 20 min at room temperature (RT) or 4°C. The plasma (supernatant) was transferred into a fresh tube and snap-frozen in liquid nitrogen for further analysis via LCMS/MS-MS.

Mouse peripheral blood mononuclear cells

Whole blood (0.7-1 mL) from mice was obtained by cardiac puncture using syringes with 10 µL K_2EDTA solution and diluted 1:1 with PBS (at RT). The diluted blood was carefully overlaid with 2 mL of Histopaque-1083® (Cat. #10831-100 mL; Sigma-Aldrich) in a 15 mL polystyrene Falcon tube and centrifuged at 400 g at RT for 30 min in a swing-out rotor. The PBMCs were aspirated, washed by addition of 800 µL of cold PBS, and centrifuged at 5,000 rpm in a micro-centrifuge for 1 min at 4°C. The supernatant was removed and the pellet resuspended in 20 - 50 µL of complete lysis buffer. Protein lysates were snap-frozen in liquid nitrogen.

Mouse hair follicles

HFs were obtained from alcohol-swabbed skin of mice, preferentially from the mouse whiskers. Individual whiskers were plucked (minimum of 40 whiskers) using sterile forceps, ensuring that the hair root was attached. Plucked whiskers were trimmed at the shafts to ~1 cm length, if required. A minimum of 40 HFs per time point/dose were collected in a 2 mL homogenization tube (Cat. #TM-625S; Tomy), containing 2 x 2.0 mm Zirconia beads (Cat. #ZB-20; Tomy) and 100 µL of complete lysis buffer. Hair was collected at the bottom of the tube by brief microcentrifugation in a Sorvall Legend Micro 21R (Cat. #75002445; Thermo Fisher Scientific) at 14,000 rpm for 5 min at 4°C.

Mouse bone marrow

Bone marrow samples were extracted from the left femur of each mouse. The femur of euthanized mice was isolated using macro-dissection, bone marrow was flushed from cleaned femurs using a syringe with an attached 29G needle filled with 2 mL of PBS at room temperature, and the eluate was collected in a sterile 15 mL centrifuge tube. The collected bone marrow cell suspension was diluted with normal saline solution to a final volume of 10 mL. The cell suspension was then centrifuged at 250 g for 8 min. The supernatant was removed and the pellet was resuspended in 200 µl of PBS. In an optional procedure, red blood cells were lyzed using red blood cell (RBC) lysis buffer. To prepare the 10x stock RBC lysis buffer 8.02 g NH_4Cl, 0.84 g $NaHCO_3$, and 0.37 g EDTA-Na_2 were dissolved in 100 mL MilliQ-water. Bone marrow cell suspension was incubated in 1x RBC lysis buffer for 10 min on ice, followed by centrifugation at 500 g for 5 min at 4°C. The supernatant was removed and the pellet was washed with 500 µL PBS (4°C). The wash was repeated a second time and the pellet lyzed in 100 µL complete lysis buffer. Bone marrow cell suspension where red blood cells were not lyzed were kept on ice until all samples were collected, followed by a centrifugation at 500 g for 5 min at 4°C, one wash with 500 µL cold PBS, and resuspension in 100 µL complete lysis buffer.

Mouse platelets

For the preparation of platelets, whole blood was obtained via cardiac puncture and collected using sodium citrate as an anticoagulant. Samples were centrifuged at 100 g for 10 min at RT without brake in a swing-out rotor. The resulting platelet-rich plasma was transferred to a separate tube. PGE_1 (Cat. #P5515; Sigma-Aldrich) was added to each sample to a final concentration of 1 µM, and samples were incubated for 5 min at RT. Sodium citrate (400 µL of a 3.8% [w/v] solution) was added for a final volume of approximately 1 mL. The preparation was centrifuged at 400 g for 10 min (without brake) at 4°C. The supernatant was discarded and the platelets were re-suspended in 20 µL complete lysis buffer.

Mouse skin

A skin sample (approximately 5 x 5 mm area) was collected from the ventral area of the animals after the fur of the mice had been cleaned using 70% ethanol and had been shaved with a hair clipper. The skin was cleaned again with 70% ethanol, followed by a rinse with neutral saline solution for 3 times before collection. Samples were obtained using sharp scissors and collected in a 2 mL homogenizing tube (Cat. #TM-625S; Tomy) containing one 5.5 mm stainless steel bead each (Cat. #SUB-55; Tomy). Approximately 200 µL of complete lysis buffer was added to each tube prior to snap-freezing in liquid nitrogen.

Human peripheral blood mononuclear cells

To prepare PBMCs from human whole blood 8 mL venous blood from healthy human volunteers was drawn into a BD Vacutainer CPT™-tube (Cat. #362761; Becton, Dickinson and Company; Franklin Lakes, NJ) containing sodium citrate, inverted 8 times, and centrifuged for 30 min at room temperature (RT) in a swing-out bucket at 1800 g within 2 h of collection. PBMCs were aspirated from the cell layer above the polyester gel using a disposable pipette and transferred into a 15 mL conical polypropylene screw cap tube containing 10 mL of PBS. PBMCs were washed 2x using 10 mL of PBS each, followed by a 15 min centrifugation at 300 g and RT. PBMCs were transferred into a 1.5 mL microcentrifuge tube in 100-200 µL of PBS after the first wash, snap-frozen in liquid nitrogen and stored at -80°C. For PD analysis in the EDDC laboratory PBMCs were quickly thawed by addition of 200 µL PBS, pre-warmed to 37°C, followed by centrifugation for 1 min at 4°C and 7,200 rpm in a microcentrifuge. After removal of the PBS, the pellet was lyzed in 25-100 µL cold complete lysis buffer, depending on the size of the pellet. Complete lysis buffer was prepared just prior to use.

Sample collections (blood draws) for PBMC isolation in the 10 mg fasted cohort (Dosing period 1[DP1]) were performed pre-dose and at the following post-dose time points: 1 h, 2 h, 4 h, 6 h, 8 h, 12 h and 24 h in the original protocol version. However, all subjects that have received the 10 mg dose in fasted state at a later time point (i.e. replacement subjects that had completed DP1 at a later stage, after DP2 and DP3, according to Protocol V5.0 dated 18 January 2017) had PBMCs collected at the following, modified time points: pre-dose, 1 h, 2 h, 4 h, 6 h, 12 h, 24 h, and 30 h post-dose. For PBMCs prepared from the 20 mg cohort (DP2) and the 10 mg fed cohort (DP3) the collection time points were the same as for the replacement subjects at 10 mg cohort: pre-dose, 1 h, 2 h, 4 h, 6 h, 12 h, 24 h, and 30 h post-dose (± 6 min).

Human hair follicles

HFs were obtained from alcohol-swabbed skin of healthy volunteers, preferentially from eyebrows or beard. Individual hairs were plucked using sterile forceps, ensuring that the hair root was attached. Plucked hairs were trimmed at the shafts to ~1 cm length, if required. A minimum of 40 HFs per time point were collected in a 2 mL homogenization tube (Cat. #TM-625S; Tomy), containing 2 x 2.0 mm zirconia beads (Cat. #ZB-20; Tomy) and 100 µL of

complete lysis buffer. HFs were collected at the bottom of the tube by brief centrifugation in a Sorvall Legend Micro 21R centrifuge (Cat. #75002445; Thermo Fisher Scientific), at 14,000 rpm, for 5 min at 4°C at the following time points: pre-dose and 1 h, 2 h, 3 h, 6 h, 12 h, and 24 h post-dose (±15 min) in all 3 DPs.

Human plasma preparation

To obtain plasma of human healthy volunteers in the Ph1 study, 3 mL of venous blood was collected using 3.0 mL Vacutainer® K_2EDTA tubes (Cat. #367856; Becton Dickinson), and blood was mixed with the anti-coagulant via gentle inversion for 8-10x. Plasma was prepared by centrifugation in a table-top centrifuge with a swing-out rotor set to room temperature (18-25°C) at a speed of 1300 g for 10 min. The supernatant was transferred into two cryotubes, snap-frozen in liquid nitrogen and stored at -80°C until transport. Blood samples for plasma preparation for PK analysis were drawn at the following times: pre-dose, 0.25 h, 0.50 h, 1 h, 1.5 h, 2 h, 3 h, 4 h, 6 h, 8 h, 12 h, 24 h, 36 h, and 48 h (± 6 min). Three additional time points (30 h, 72 h [both ± 6 min], and 144 h [± 30 min]) were added in Protocol V5.0 dated 18 January 2017 and were included for HVs dosed in DP2 and DP3 as well as for two replacement subjects that had their DP1 (10 mg fasted) after completing DP2 and/or DP3.

Human skin punch biopsies

Skin biopsies were obtained from consented subjects in DP3 only under local anesthesia with 1% lidocaine for injection, applied subcutaneously, using a 23G needle. A skin punch from the back of the HVs was obtained using a 3 mm diameter skin punch tool after disinfection of the area with an alcohol swab. The skin core was placed in a 2 mL homogenizing tube containing 8 x 2.0 mm zirconia beads (Cat. #TM-625S with Cat. #ZB-20; Tomy) using sterile forceps and was snap-frozen in liquid nitrogen and stored below -65°C until homogenization. Two skin biopsies were collected from each subject, one on the day before dosing (D-1) and another one 1.5 h (± 30 min) post-dose. All of the DP3 subjects were supposed to receive 10 mg ETC-206 after a high-fat meal. However, the subjects were accidentally randomized to active and control and therefore 2/9 subjects had a skin biopsy collected after placebo treatment.

Extraction and bioanalysis of ETC-206 in mouse samples

Extraction was done at the Biological Resource Centre, Singapore, in 20 µL of the diluted tissue using acetonitrile with addition of imipramine (20 ng/mL) as internal standard [IS], Samples (25 µL of supernatant) were precipitated by adding 225 µL of acetonitrile containing IS, vortexed for 10 min at 2,000 rpm in a high-speed microplate shaker, centrifuged for 30 min at 4°C and 4,000 g in a 96 well plate, and injected into UPLC-MS/MS. Bioanalysis of ETC-206 using mass spectrometry from mouse studies was performed using UPLC–MS/MS (Waters Acquity I-Class UPLC system coupled with a Xevo TQ-S Triple Quadrupole Mass spectrometer (both Waters Corporation, Milford, MA). To separate ETC-206 and the IS the chromatography was performed using an Acquity BEH C18 column (2.1 mm × 75 mm; 1.7 µm particle size) coupled with an UPLC column in-line stainless steel filter kit (0.2 µm filter). A gradient method was used for chromatographic separation with the mobile phase consisting of A (10 mM ammonium acetate in deionized water), and B (acetonitrile containing 0.1% formic acid) with the following composition: 0–0.2 min (95% A), 0.2–1.7 min (25 % A), 1.7–1.8 min (95% A), 1.8–2.0 min (95% A). The mobile phase flow rate was 0.40 mL/min. The total run time was 2 min with 2 µL injection volume at a column temperature set to 40°C. For the MS analysis the UPLC flow was analyzed using the Xevo TQ-S (Waters Corporation). Electro-spray ionization (ESI^+) was used as ionization source, detection was by multiple reaction monitoring (MRM) with following transitions for ETC-206 and the internal standard: m/z

409.17→324.12 and m/z 281.16→58.11 respectively. The ESI–MS/MS parameters settings were as follows: capillary voltage: 4 kV; cone voltage, 90V; source temperature, 135°C; desolvation temperature: 250°C; gas flow to cone 150 L/h, desolvation gas flow: 850 L/h (nitrogen). The collision gas was argon and was introduced into the collision cell at a flow rate of 0.15 mL/min. Data acquisition was done using Masslynx 4.1 software and processed using TargetLynx™ Application Manager (both Waters Corporation).
